# Atopic dermatitis/eczema phenotypes and their association with food allergy: a nationwide birth cohort study in Japan

**DOI:** 10.1007/s00431-026-07192-y

**Published:** 2026-06-22

**Authors:** Yasutaka Kuniyoshi

**Affiliations:** Department of Social Services and Healthcare Management, International University of Health and Welfare, 2600-1 Kitakanemaru, Otawara, Tochigi 324-8501 Japan

**Keywords:** Atopic dermatitis, Birth cohort, Food allergy, Longitudinal studies, Phenotypes

## Abstract

Atopic dermatitis (AD) is a heterogeneous disease often preceding food allergy (FA). However, it remains unclear how different developmental trajectories of AD/eczema influence the risk of FA. This study aimed to characterize longitudinal AD/eczema phenotypes from infancy to early adolescence and evaluate their specific associations with FA risk. We analyzed data from the Longitudinal Survey of Newborns in the 21st Century in Japan, including 23,767 participants followed from age 0.5 to 12 years. Distinct AD/eczema phenotypes were identified using group-based trajectory modeling derived from healthcare visit histories. Multivariable logistic regression examined the association between AD/eczema phenotypes and cumulative FA healthcare visit history, adjusting for sociodemographic factors and asthma comorbidity. Five AD/eczema phenotypes were identified: "Early-onset transient" (6.4%), "Early-onset persistent" (23.2%), "Toddler-onset persistent" (17.3%), "Late-onset" (2.1%), and "No/minimal symptoms" (51.0%). Compared with the "No/minimal symptoms" group, all AD/eczema phenotypes were associated with increased FA risk. The "Early-onset transient" (adjusted odds ratio [aOR], 2.33; 95% CI, 1.98–2.74) and "Early-onset persistent" (aOR, 2.33; 95% CI, 2.10–2.59) groups showed the strongest associations. The "Late-onset" phenotype was also associated with increased risk (aOR, 1.42; 95% CI, 1.04–1.93), with elevated risk observed in early childhood preceding the peak of overt skin symptoms.

*Conclusion*: Distinct developmental trajectories of AD/eczema are differentially associated with FA risk. While early-onset phenotypes confer the highest risk, the elevated risk in "Late-onset" trajectories before peak symptoms suggests shared underlying susceptibility or subclinical pathology. Monitoring FA development is important across all clinical trajectories of AD/eczema.

**Table:** 

**What is Known:** • *Atopic dermatitis (AD) is a primary precursor for the atopic march, but how different longitudinal trajectories influence food allergy (FA) risk remains unclear.* • *Early-onset persistent AD is considered to pose the highest risk for FA.*
**What is New:** • *Early-onset transient AD/eczema carries a high FA risk comparable to persistent cases, indicating that timing of onset is more critical than disease duration for FA development.* • *Late-onset AD/eczema trajectories show elevated FA risk in early childhood preceding peak skin symptoms, suggesting shared underlying susceptibility or subclinical pathology.*

**Supplementary Information:**

The online version contains supplementary material available at 10.1007/s00431-026-07192-y.

## Introduction

Atopic dermatitis (AD), a chronic inflammatory skin disease, affects approximately 20% of children worldwide [1, 2]. It also serves as a primary precursor for the “atopic march,” predisposing patients to the development of food allergy (FA). AD is a heterogeneous condition with distinct clinical and pathophysiological subtypes [2]. Identifying these specific developmental phenotypes is essential for optimizing clinical management, understanding disease pathogenesis, and stratifying the risk of allergic comorbidities [3, 4].

Previous longitudinal studies have identified several AD phenotypes defined by age of onset and disease persistence (e.g., “transient” and “persistent”) [5–10]. These distinct trajectories are associated with diverse genetic backgrounds, such as filaggrin gene mutations [7, 11] and varying immunologic profiles [12]. However, current clinical risk assessment commonly depends on cross-sectional evaluation of disease severity. This static approach does not account for the longitudinal trajectory of skin symptoms and may overlook critical developmental periods when the skin is most susceptible to percutaneous sensitization.

Although AD is widely recognized as an initial step in the atopic march [13], it remains unclear whether all AD phenotypes equally contribute to this progression. Furthermore, the temporal association between skin inflammation and food sensitization is complex. While previous studies have primarily focused on a linear “skin-to-food” progression driven by clinically apparent dermatitis, it remains unknown whether FA risk depends entirely on the visible onset and duration of eczema, or if it also involves subclinical barrier defects or systemic immune dysregulation preceding overt skin symptoms [14].

Therefore, this study aimed to characterize distinct AD trajectories and determine their specific associations with FA risk using data from a large, nationwide birth cohort in Japan. We hypothesized that different AD trajectories carry varying FA risks, with the “early-onset persistent” phenotype posing the highest risk. Moreover, we sought to elucidate the temporal sequence between distinct skin symptom trajectories and the initiation of FA healthcare visits, thereby providing insights into the developmental pathways and temporal dynamics of the atopic march.

## Materials and methods

### Study design, setting, and participants

This study represents a secondary analysis of data from the Longitudinal Survey of Newborns in the 21st Century, a nationwide birth cohort study conducted by the Japanese Ministry of Health, Labour and Welfare [15]. Follow-up of this cohort is ongoing. The cohort encompassed infants born between May 10 and May 24, 2010 (*n* = 43,767). The surveys were primarily administered via mail-based self-administered questionnaires. From age 10 years onward, online response options were also introduced to improve response rates. To account for address changes during follow-up, participants were required to report their new addresses to a dedicated contact point, and survey records were updated accordingly to ensure questionnaires reached their current residence. The initial survey was conducted at the age of 6 months, and yearly follow-up surveys were conducted afterward. A total of 38,554 participants responded to the initial survey (response rate, 88.0%). The return of a completed questionnaire was considered to be an implicit consent for participation.

Data from an initial survey at 0.5 years and 11 follow-up surveys, administered at ages 1.5, 2.5, 3.5, 4.5, 5.5, 7.0, 8.0, 9.0, 10.0, 11.0, and 12.0 years, were utilized for this analysis. As each questionnaire evaluated the healthcare visit history over the preceding year, the analysis effectively covered the clinical course from ages 0.5 to 12.0 years, with the exception of the period between 5.5 and 7.0 years where no survey was conducted. Participants with ≥ 4 missing responses regarding outpatient visits for AD or eczema across the 11 follow-up surveys were excluded from the analysis. This threshold was determined a priori to ensure sufficient data completeness for reliable trajectory group assignment. All procedures conformed to the ethical standards of the Helsinki Declaration of 1975, as revised in 2013. The study protocol was approved by the Ethics Committee of the International University of Health and Welfare (Approval No. 25-TC-107). This study was reported in accordance with the Strengthening the Reporting of Observational Studies in Epidemiology guidelines [16].

### Measurements

#### AD/eczema phenotype

The primary exposure was membership in distinct AD/eczema phenotype groups. Phenotypes were determined using parental responses to questionnaires administered at ages 1.5, 2.5, 3.5, 4.5, 5.5, 7.0, 8.0, 9.0, 10.0, 11.0, and 12.0 years. As each questionnaire evaluated the healthcare visit history over the preceding year, the analysis effectively covered the clinical course from ages 0.5 to 12 years. A history of healthcare visits for AD or eczema was confirmed if a parent responded “yes” to the question “Have you visited a hospital or clinic for any illness or injury in the past year?” and selected “AD” or “eczema” from a predefined list of conditions (Text S1).

#### Food allergy

The primary outcome was a history of healthcare visits for FA, which was confirmed if a parent responded “yes” to the question “Have you visited a hospital or clinic for any illness or injury in the past year?” and selected “FA” from a predefined list of conditions (Text S1).

#### Covariates

The following variables were included as covariates in the multivariable logistic regression analyses to adjust for potential confounding: Sex (male or female), birth type (singleton or multiple), gestational age (≥ 37 or < 37 weeks), birth weight (< 2,500 or ≥ 2,500 g), number of elder siblings at the age of 6 months (0 or ≥ 1), feeding practice (exclusive breastfeeding, partial breastfeeding, or exclusive formula feeding), daycare attendance at the age of 6 months (attending or not attending), household income (< 2.50, 2.50–4.99, 5.00–7.49, 7.50–9.99, or ≥ 10.00 million JPY), maternal and paternal educational attainment (junior high school, high school, university/junior college/vocational school, or others), maternal and paternal smoking status at the age of 6 months (smoking or nonsmoking), and history of healthcare visits for asthma (yes or no) at each survey time point from ages 1.5 to 12 years. Allergic rhinitis was not included as a covariate because the survey did not include allergic rhinitis as a standalone response option in the predefined list of conditions. Feeding practices were classified according to the detailed responses in the parental surveys [17].

#### Sample size

As this study represents a secondary analysis of a large, nationwide cohort, no formal a priori sample-size calculation was performed. All eligible participants from the original cohort were included in the analysis.

#### Statistical analysis

Baseline characteristics were compared among the identified AD/eczema phenotypes using the chi-squared test for categorical variables. Furthermore, the characteristics of participants included in and excluded from the final analysis were compared to evaluate for potential selection bias.

Group-based trajectory modeling (GBTM) was employed to identify distinct individual subgroups following similar AD/eczema trajectories from the ages of 1.5–12 years [18]. Models were fitted with two to five groups and linear, quadratic, and cubic polynomial forms. Based on previous longitudinal AD trajectory studies that typically identified four to five distinct phenotypes [5–10], and considering the balance between statistical model fit and clinical interpretability, we decided a priori to evaluate models with two to five groups. The optimal trajectory model was chosen based on the lowest Bayesian information criterion, average posterior probability ≥ 0.80 for each group, adequate group size, and clinical interpretability. Each participant was assigned to the trajectory group corresponding to their highest probability of membership. The prevalence of FA was stratified by AD/eczema phenotype and plotted to visually assess temporal trends.

Subsequently, multiple imputations were performed using chained equations to handle missing data in covariates and the FA outcome variable after group assignment. A total of 100 imputed datasets were generated, each having 20 iterations. Logistic and polytomous logistic regressions were employed for binary and categorical variables, respectively. The final estimates were combined using Rubin’s rules.

Multivariable logistic regression analyses were conducted to examine the association between AD/eczema phenotypes and a cumulative history of healthcare visits for FA from ages 0.5 to 12 years using the “No/minimal symptoms” phenotype as the reference category. The covariates listed in the Covariates subsection (sex, birth type, gestational age, birth weight, number of elder siblings, feeding practice, daycare attendance, household income, parental educational attainment, parental smoking status, and cumulative asthma healthcare visit history) were included in these models to adjust for potential confounding. In the subgroup analyses, two separate outcomes were evaluated: a cumulative history of healthcare visits for FA during early childhood (age 0.5–5.5 years) and during school age (age 6–12 years). In these models, the cumulative history of healthcare visits for asthma used for adjustment was defined based on surveys from ages 1.5 to 5.5 years and 1.5 to 12 years, respectively. In addition, a complete-case analysis was conducted as a sensitivity analysis, excluding participants with missing data.

All analyses were conducted using the R software version 4.2.3 (R Foundation for Statistical Computing, Vienna, Austria) and specific packages, including “tidyverse” for data manipulation, “mice” for multiple imputation, “gbmt” for GBTM modeling, and “glm” function for multivariable logistic regression.

## Results

### Participant characteristics

**Fig. 1:**
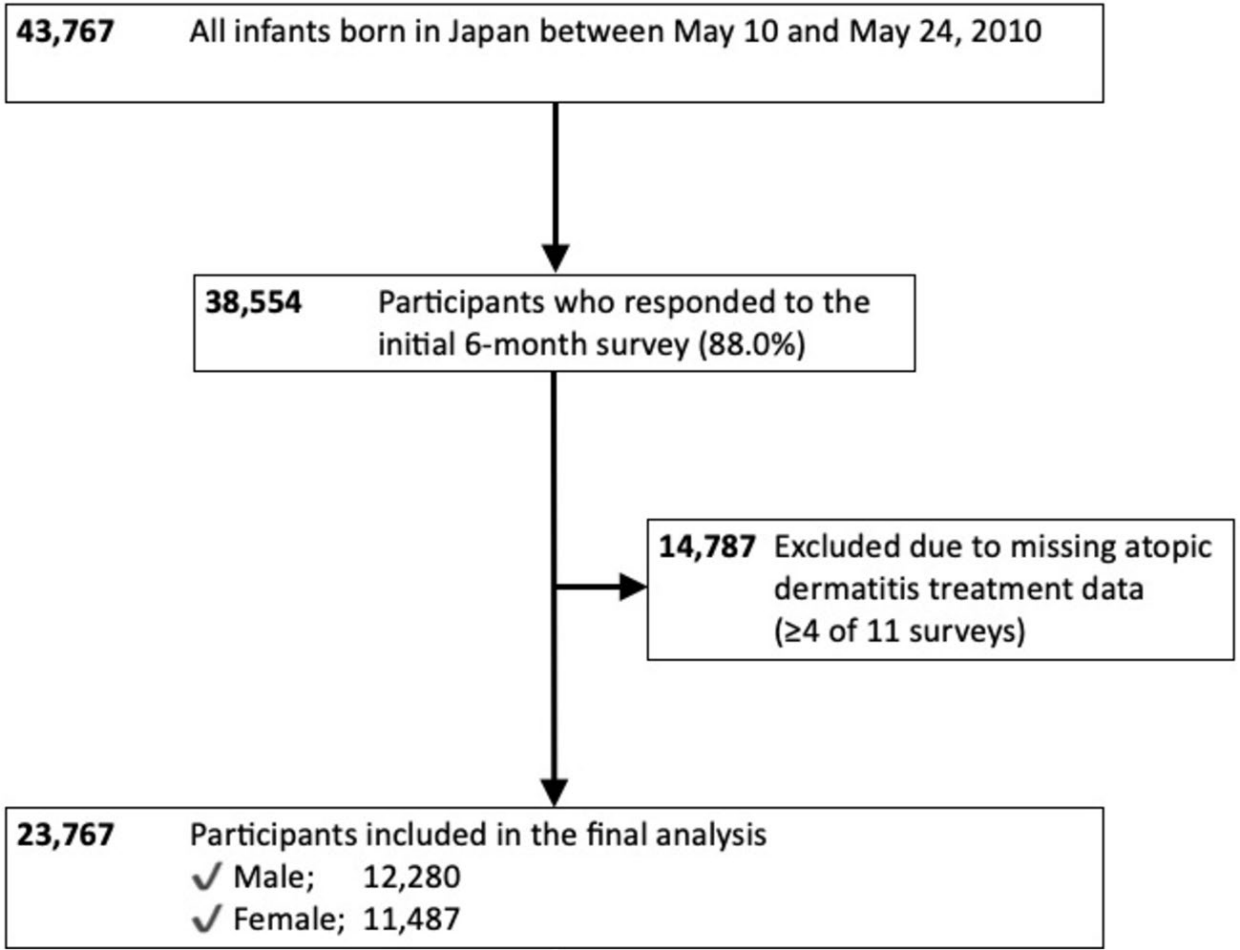
Flowchart of participant selection

Figure 1 presents a flowchart of the study cohort selection process. Of the 38,554 participants who responded to the initial survey, 14,787 were excluded due to missing information on the history of healthcare visits for AD/eczema. Finally, 23,767 participants were included in the analysis. Of the 23,767 participants, 12,280 (51.7%) were male and 11,487 (48.3%) were female. Compared with the participants included in the analysis, those who were excluded showed a higher likelihood of having mothers who smoked, lower parental educational attainment, and lower household income (Table S1). Missing data increased over time, ranging from 16.9% at age 1.5 years to 46.8% at age 12 years (Table S2). The cumulative prevalence rates of FA and asthma healthcare visits by 12 years of age were 18.5% (2,508/11,038) and 28.5% (4,103/14,386), respectively. Participants with FA data had higher socioeconomic status and different AD/eczema phenotype distributions compared to those with missing FA data (Table S3). The cohort’s baseline characteristics, stratified by AD/eczema phenotype, are presented in Table 1 and Table S4. Notably, the prevalence of asthma healthcare visits was highest in the 'Early-onset persistent' (30%), 'Toddler-onset persistent' (31%), and 'Late-onset' (31%) phenotypes compared with the 'No/minimal symptoms' group (27%), reflecting the strong comorbidity between persistent AD/eczema and asthma. In contrast, feeding practices showed only minor variation across phenotypes (exclusive breastfeeding: 34%–39%; mixed feeding: 59%–63%), suggesting that infant feeding patterns alone do not substantially determine AD/eczema trajectory development. Regarding sociodemographic factors, the distribution was relatively balanced across phenotypes, with the 'No/minimal symptoms' group showing slightly lower household income (≥ 10.00 million JPY: 9.0%) compared with other phenotypes (9.2%–11%).

**Table 1:** Baseline characteristics of the study participants according to atopic dermatitis/eczema phenotypes^a^

	Early-onset transient N = 1,522	Early-onset persistent N = 5,504	Toddler-onset persistent N = 4,113	Late-onset N = 502	No/minimal symptoms N = 12,126	*P*-value^b^
Sex						< 0.001
Female	683 (45%)	2,660 (48%)	2,087 (51%)	254 (51%)	5,803 (48%)	
Male	839 (55%)	2,844 (52%)	2,026 (49%)	248 (49%)	6,323 (52%)	
Birth weight						
< 2,500 g	116 (7.6%)	520 (9.4%)	373 (9.1%)	48 (9.6%)	1,104 (9.1%)	
≥ 2,500 g	1,406 (92%)	4,984 (91%)	3,740 (91%)	454 (90%)	11,019 (91%)	
Missing	0	0	0	0	3	
Gestational age						0.5
< 37 weeks	67 (4.4%)	296 (5.4%)	200 (4.9%)	24 (4.8%)	628 (5.2%)	
≥ 37 weeks	1,455 (96%)	5,207 (95%)	3,913 (95%)	478 (95%)	11,496 (95%)	
Missing	0	1	0	0	2	
Number of elder siblings at 6 months						< 0.001
≥ 1	673 (44%)	2,753 (50%)	2,075 (50%)	268 (53%)	6,430 (53%)	
0	849 (56%)	2,751 (50%)	2,038 (50%)	234 (47%)	5,695 (47%)	
Missing	0	0	0	0	1	
Birth type						0.6
Multiple	25 (1.6%)	91 (1.7%)	61 (1.5%)	7 (1.4%)	220 (1.8%)	
Singleton	1,497 (98%)	5,413 (98%)	4,052 (99%)	495 (99%)	11,906 (98%)	
Maternal smoking status at 6 months						0.018
Nonsmoking	1,467 (97%)	5,302 (97%)	3,945 (96%)	483 (97%)	11,563 (96%)	
Smoking	53 (3.5%)	189 (3.4%)	160 (3.9%)	17 (3.4%)	537 (4.4%)	
Missing	2	13	8	2	26	
Paternal smoking status at 6 months						0.074
Nonsmoking	964 (64%)	3,506 (65%)	2,574 (64%)	310 (63%)	7,441 (62%)	
Smoking	536 (36%)	1,925 (35%)	1,466 (36%)	186 (38%)	4,477 (38%)	
Missing	22	73	73	6	208	
Maternal educational attainment						
Junior high school	42 (2.8%)	154 (2.9%)	122 (3.0%)	9 (1.9%)	385 (3.3%)	
High school	591 (39%)	2,196 (41%)	1,674 (42%)	214 (44%)	5,431 (46%)	
University/junior college/vocational school	864 (58%)	3,024 (56%)	2,211 (55%)	263 (54%)	5,977 (51%)	
Others	0 (0%)	19 (0.4%)	14 (0.3%)	0 (0%)	31 (0.3%)	
Missing	25	111	92	16	302	
Paternal educational attainment						
Junior high school	53 (3.6%)	231 (4.3%)	217 (5.5%)	21 (4.4%)	629 (5.4%)	
High school	602 (41%)	2,194 (41%)	1,650 (42%)	205 (43%)	5,238 (45%)	
University/junior college/vocational school	821 (55%)	2,890 (54%)	2,088 (53%)	251 (52%)	5,765 (49%)	
Others	4 (0.3%)	17 (0.3%)	8 (0.2%)	3 (0.6%)	35 (0.3%)	
Missing	42	172	150	22	459	
Daycare attendance at 6 months						0.4
Attending	53 (3.5%)	173 (3.1%)	112 (2.7%)	12 (2.4%)	384 (3.2%)	
Not attending	1,469 (97%)	5,331 (97%)	4,000 (97%)	490 (98%)	11,741 (97%)	
Missing	0	0	1	0	1	
Household Income						< 0.001
< 2.50 million JPY	45 (3.1%)	135 (2.6%)	123 (3.2%)	17 (3.5%)	467 (4.1%)	
2.50–4.99 million JPY	471 (33%)	1,646 (32%)	1,316 (34%)	152 (32%)	3,915 (35%)	
5.00–7.49 million JPY	532 (37%)	1,928 (37%)	1,410 (37%)	187 (39%)	4,156 (37%)	
7.50–9.99 million JPY	251 (17%)	894 (17%)	649 (17%)	75 (16%)	1,727 (15%)	
≥ 10.00 million JPY	142 (9.9%)	566 (11%)	354 (9.2%)	49 (10%)	1,019 (9.0%)	
Missing	81	335	261	22	842	
Feeding practice						< 0.001
Exclusive breastfeeding	517 (34%)	1,945 (35%)	1,405 (34%)	195 (39%)	4,281 (35%)	
Exclusive formula feeding	54 (3.6%)	126 (2.3%)	100 (2.4%)	9 (1.8%)	409 (3.4%)	
Mixed feeding	942 (62%)	3,410 (62%)	2,584 (63%)	292 (59%)	7,371 (61%)	
Missing	9	23	24	6	65	
Asthma healthcare visit history						< 0.001
No	711 (74%)	2,551 (70%)	1,944 (69%)	221 (69%)	4,856 (73%)	
Yes	256 (26%)	1,109 (30%)	854 (31%)	97 (31%)	1,787 (27%)	
Missing	555	1,844	1,315	184	5,483	
Food allergy healthcare visit history						< 0.001
No	725 (76%)	2,667 (76%)	2,090 (78%)	258 (84%)	5,298 (87%)	
Yes	227 (24%)	838 (24%)	601 (22%)	48 (16%)	794 (13%)	
Missing	570	1,999	1,422	196	6,034	

^a^Data are expressed as numbers (%) for categorical variables. ^b^*P* values were calculated to evaluate differences across the AD/eczema phenotypes using the chi-squared test for categorical variables

AD, atopic dermatitis; FA, food allergy

### Identification of AD/eczema phenotypes

**Fig. 2:**
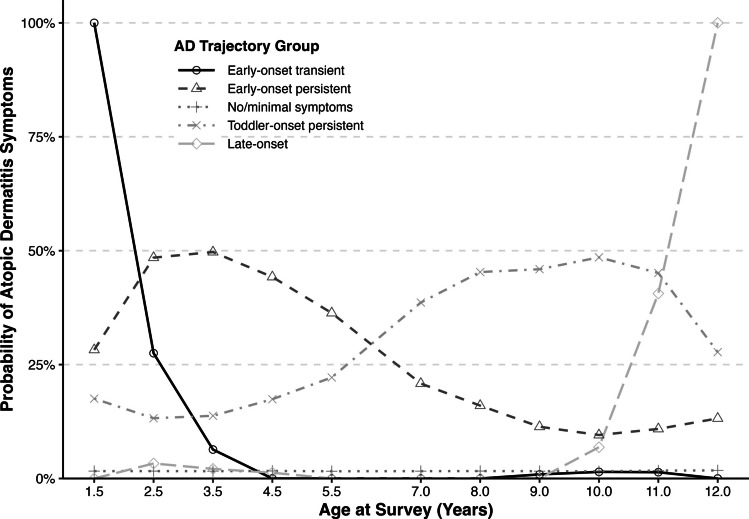
Longitudinal trajectories of atopic dermatitis/eczema phenotypes from ages 0.5 to 12 years. Trajectories were identified via GBTM modeling based on parent-reported healthcare visits from surveys administered at ages 1.5, 2.5, 3.5, 4.5, 5.5, 7.0, 8.0, 9.0, 10.0, 11.0, and 12.0 years. Data points denote the prevalence of healthcare visits for AD/eczema reported at each survey age, indicating the healthcare visit history during the preceding year (e.g., data at 1.5 years cover the period from 0.5 to 1.5 years)

Five distinct AD/eczema phenotypes from the age of 0.5 to 12 years, labeled according to their shape, were identified via GBTM (Table S5, Figure S1): (1) “Early-onset transient” (*n* = 1,522; 6.4%), (2) “Early-onset persistent” (*n* = 5,504; 23.2%), (3) “Toddler-onset persistent” (*n* = 4,113; 17.3%), (4) “Late-onset” (*n* = 502; 2.1%), and (5) “No/minimal symptoms” (*n* = 12,126; 51.0%). Figure 2 presents the observed probability of AD/eczema healthcare visits over time among participants assigned to each phenotype group. “Early-onset transient” was marked by a high probability of symptoms at the age of 1.5 years, which then sharply declined. “Early-onset persistent” was characterized by a high symptom probability throughout early childhood. “Toddler-onset persistent” was characterized by a peak in the probability of symptoms at the age of approximately 3.5 years, followed by a gradual decline. “Late-onset” exhibited a distinct increase in symptom probability during late childhood and early adolescence. “No/minimal symptoms” consistently demonstrated a very low symptom probability throughout the follow-up period.

### Temporal trends of FA healthcare visit history

**Fig. 3:**
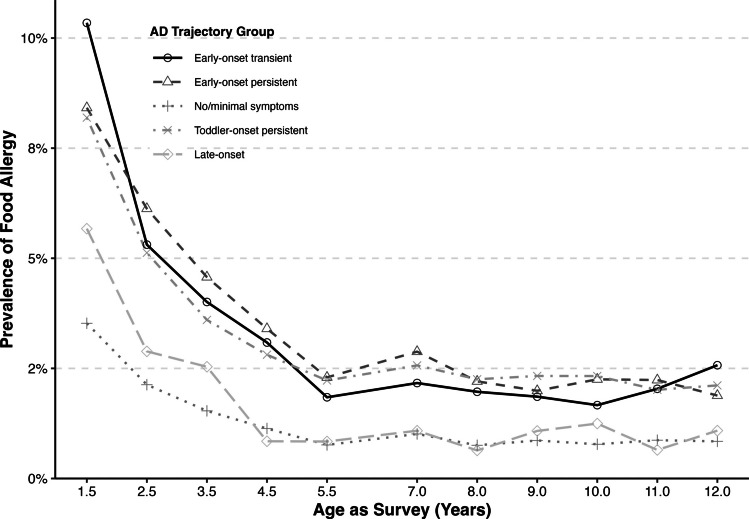
Temporal trends in the prevalence of food allergy by atopic dermatitis/eczema phenotype. The graph presents the percentage of participants with a parent-reported history of the healthcare visits for food allergy stratified by the five atopic dermatitis/eczema phenotypes defined in Fig. 2. Surveys were administered at ages 1.5, 2.5, 3.5, 4.5, 5.5, 7.0, 8.0, 9.0, 10.0, 11.0, and 12.0 years. Data points denote the prevalence of healthcare visits reported at each survey age, indicating the history during the preceding year (e.g., the prevalence at 1.5 years covers the period from 0.5 to 1.5 years)

Figure 3 presents the longitudinal trends in the prevalence of FA healthcare visit history stratified by AD/eczema phenotype from ages 0.5 to 12 years. The groups exhibited distinct temporal patterns. At age 1.5 years, the prevalence was highest in the 'Early-onset transient' phenotype (approximately 10%), followed closely by the 'Early-onset persistent' and 'Toddler-onset persistent' phenotypes (both approximately 9%). Although the “Early-onset transient” group exhibited a sharp decline after age 3 years, paralleling the remission of their skin symptoms, the “Early-onset persistent” and “Toddler-onset persistent” groups maintained a higher prevalence throughout childhood compared with the “No/minimal symptoms” group, which started at approximately 3% at age 1.5 years and remained the lowest across all time points.

### Association between AD/eczema phenotypes and FA

**Fig. 4:**
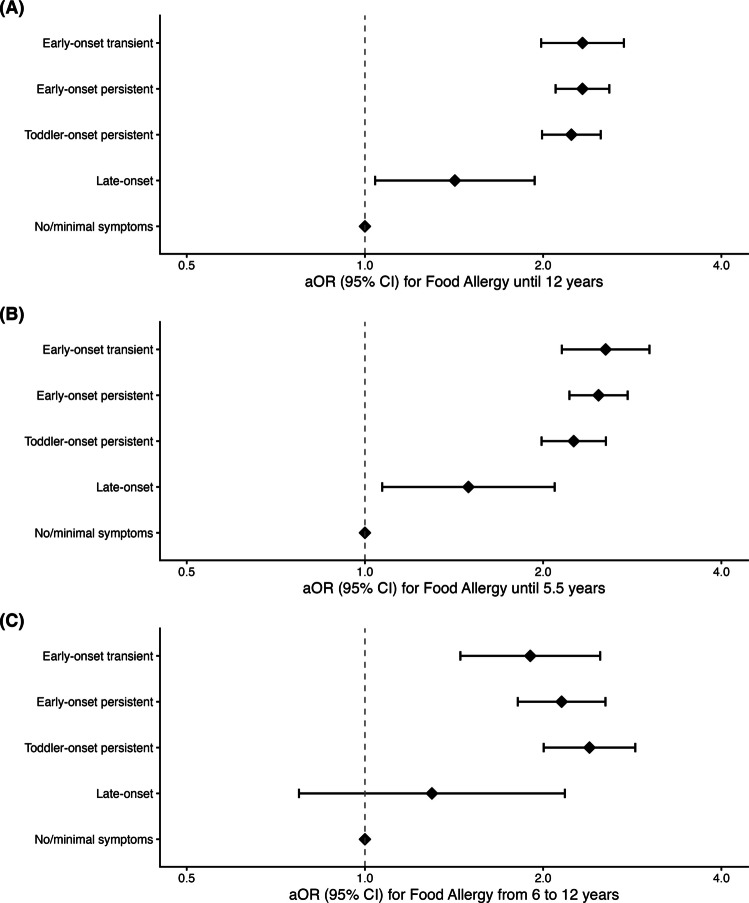
Association of atopic dermatitis/eczema phenotypes with history of healthcare visits for food allergy. Forest plots display aORs (points) and 95% CIs (horizontal lines) derived from multivariable logistic regression models. Data were handled using multiple imputation. The “No/minimal symptoms” phenotype serves as the reference group. Models were adjusted for sociodemographic factors, perinatal characteristics, and cumulative history of healthcare visits for asthma. (**A**) Cumulative history of healthcare visits for food allergy from ages 0.5 to 12 years. (**B**) Cumulative history of healthcare visits for food allergy during early childhood (ages 0.5–5.5 years). (**C**) Cumulative history of healthcare visits for food allergy during school age (ages 6–12 years)

Figure 4 presents the adjusted odds ratios (aORs) for the association between AD/eczema phenotypes and a history of healthcare visits for FA. In the primary analysis encompassing the entire follow-up period (0.5–12 years; Fig. 4A), all the identified AD/eczema phenotypes showed an association with an increased risk of FA compared with the “No/minimal symptoms” reference group. The “Early-onset transient” and “Early-onset persistent” phenotypes demonstrated the strongest associations (aOR, 2.33 [95% CI, 1.98–2.74] and 2.33 [95% CI, 2.10–2.59], respectively), followed by the “Toddler-onset persistent” phenotype (aOR, 2.23; 95% CI, 1.99–2.50). The “Late-onset” phenotype also showed an association with healthcare visit history for FA, although the magnitude of the risk was attenuated (aOR, 1.42; 95% CI, 1.04–1.93).

Subgroup analyses stratified by age revealed distinct temporal patterns. For FA healthcare visit history during early childhood (0.5–5.5 years; Fig. 4B), positive associations were found across all phenotypes, including “Late-onset” (aOR, 1.50; 95% CI, 1.07–2.09). However, for FA healthcare visit history during school age (6–12 years; Fig. 4C), the early- and toddler-onset phenotypes maintained strong associations (aOR range, 1.90–2.39), whereas the “Late-onset” phenotype exhibited a weakened association, with confidence intervals (CIs) including the null value (aOR, 1.30; 95% CI, 0.77–2.18).

Finally, a complete-case analysis was conducted, excluding participants with missing data (n = 11,901), to evaluate the robustness of our findings (Figure S2, Table S6). The results were generally consistent with those of the primary analysis conducted using multiple imputation for the Early-onset transient, Early-onset persistent, and Toddler-onset persistent phenotypes. However, the Late-onset group lost statistical significance in the complete-case analysis, with the confidence interval including the null value, likely due to the substantial reduction in sample size (from 502 to 268 participants, a 47% reduction).

## Discussion

The principal finding of this nationwide birth cohort study that included 23,767 children is that the distinct developmental trajectories of AD/eczema are differentially associated with an FA risk over the first 12 years of life. Although early-onset phenotypes exhibited the highest risk, consistent with the classical “atopic march,” our analysis revealed that all the phenotype groups exhibited an increased FA risk compared with children with no or minimal symptoms. Even among children in the “Late-onset” group, an elevated FA risk was observed in early childhood, preceding the peak of their cutaneous symptoms. These results suggest that all identified clinical trajectories of AD/eczema may indicate susceptibility to systemic allergic progression.

Consistent with previous longitudinal studies [5, 7], our study confirmed that early-onset phenotypes exhibited a positive association with FA risk. This finding supports the conceptual framework that early-life skin inflammation serves as a risk factor for allergic comorbidities such as FA, which represents a key component of the atopic march [4]. However, as we did not model the longitudinal trajectories of asthma or allergic rhinitis in this study, our analysis does not capture the full sequential progression of the atopic march. The observation that the “Early-onset transient” phenotype exhibited a risk magnitude comparable to that of the “Early-onset persistent” phenotype suggests that the timing of disease onset during a critical developmental period is more strongly associated with FA risk than the duration of clinical symptoms. These results suggest that once the allergic trajectory associated with early-life eczema is established, susceptibility to FA may persist independently of subsequent cutaneous remission.

Our analysis revealed that the “Late-onset” phenotype was associated with an increased FA risk during early childhood (0.5–5.5 years). This association precedes the peak prevalence of overt cutaneous symptoms in this trajectory. Although this finding is consistent with hypotheses regarding subclinical barrier defects [14] or systemic immune dysregulation, the potential influence of detection bias and reverse causality must also be considered. Children with early-onset FA often require frequent medical visits for dietary management, giving physicians more opportunities to assess the skin. Consequently, mild eczema that might otherwise be overlooked may be recognized and treated as AD in this group. Moreover, this temporal sequence adds complexity to the “skin-to-food” progression of the atopic march. Rather than overt dermatitis being a prerequisite for sensitization, FA and AD/eczema in the “Late-onset” trajectory may represent distinct comorbidities arising from a shared genetic predisposition but emerging on different timelines [4, 12]. During late childhood (6–12 years), the association with FA risk in this group attenuated, with broadened CIs indicating increased statistical uncertainty. It remains unclear whether the allergic comorbidity in this phenotype resolves over time or if the smaller subgroup size limits the power to detect an ongoing association.

The biological plausibility of our findings is supported by the “dual-allergen exposure” hypothesis. This model proposes that low-dose skin exposure to food allergens through an impaired skin barrier promotes allergic sensitization and Th2-biased immune responses, whereas early oral consumption encourages immune tolerance [19, 20]. Although our study was based on healthcare visit history rather than objective severity metrics, the ongoing need for medical intervention in the “Early-onset persistent” group may reflect prolonged disease activity and related skin barrier dysfunction. This interpretation is consistent with recent evidence demonstrating that objective disease severity is directly correlated with the frequency of FA comorbidity [21, 22]. Moreover, this barrier-mediated pathway may be particularly relevant in Asian populations [23], given the higher prevalence of specific genetic variants, such as filaggrin mutations, and potentially distinct environmental exposures, although variations in diagnostic practices and healthcare-seeking behaviors must also be considered. However, although “skin-to-food” progression is a dominant pathway, developmental trajectories appear to be heterogeneous [4]. Not all patients follow a uniform course, suggesting that the observed clinical phenotypes reflect a complex underlying pathology with varying degrees of barrier dysfunction and allergic susceptibility.

From a clinical perspective, our findings highlight the potential value of AD/eczema phenotypes for risk stratification while also emphasizing the challenge of making early prediction. The “persistent” or “Late-onset” trajectory can only be definitively confirmed longitudinally. For these infants, increased vigilance regarding FA development is required, not to encourage indiscriminate screening but to facilitate safe and timely dietary introduction under appropriate management. Moreover, regarding prevention, recent evidence on maintaining skin barrier integrity using interventions such as emollient use has been inconsistent [24–27]. Such an inconsistency may partially stem from the heterogeneity of AD/eczema phenotypes included in trials, where the advantages in high-risk groups could be diminished. However, as the “persistent” trajectory is currently discernible only retrospectively, a shift from universal to targeted prevention initially requires the establishment of reliable prognostic biomarkers to identify these high-risk infants at disease onset.

This study has several notable strengths. First, the utilization of a large, nationwide birth cohort offers substantial statistical power. Second, the long-term longitudinal design, spanning from infancy to early adolescence, enabled the delineation of distinct developmental trajectories. This approach more effectively captures the dynamic nature of allergic disease progression compared with a cross-sectional design, although it is acknowledged that the resolution is limited to the specific survey time points at approximately 1-year intervals. Third, the application of GBTM modeling allowed a more detailed understanding of the clinical course of AD/eczema compared with traditional single-time-point assessments, which often obscure the impact of disease duration and onset timing.

This study has several limitations. First, the exclusion of 14,787 participants (38.4%) with ≥ 4 missing AD/eczema assessments may introduce selection bias, as these participants systematically differed in terms of key socioeconomic variables. Although our models were adjusted for these factors, residual bias owing to this attrition cannot be ruled out. Specifically, if participants with higher AD/eczema burden or socioeconomic challenges were disproportionately excluded, our effect estimates may conservatively reflect the true associations. Nevertheless, the consistency between our imputed and complete-case analyses supports the internal validity of our findings within the analyzed cohort. To enhance transparency, we have added Table S2 detailing the distribution of missing assessments by survey age and by the number of missing time points per participant. Second, reliance on parent-reported healthcare visit history for exposure and outcome potentially introduces potential measurement error and information bias. This approach lacks objective severity measures and strict diagnostic criteria. For AD/eczema, the inclusion of “eczema” might introduce misclassification by capturing nonatopic conditions, whereas mild cases managed with over-the-counter remedies might be overlooked. As regards FA, the data likely include cases of unconfirmed diagnoses or anxiety-driven visits. Additionally, we cannot definitively determine whether all reported FA cases represent IgE-mediated food allergy, which is typically considered in the atopic march framework, or include non-IgE-mediated conditions such as food protein-induced enterocolitis syndrome. Although the questionnaire did not include separate options for non-IgE-mediated conditions, this limitation should be considered when interpreting our findings in the context of the atopic march. FA also exhibited a high missingness rate (43%, 10,221/23,767), with missingness strongly correlated with missingness in other variables. Participants with FA information had higher socioeconomic status compared to those with missing FA data (Table S6), indicating that missingness was related to observed characteristics. While our multiple imputation approach accounts for these differences under the missing-at-random assumption, we cannot exclude the possibility of residual selection bias. Moreover, although nondifferential misclassification typically biases estimates toward the null [28], the potential for dependent misclassification must be acknowledged; parents of children with severe skin symptoms may be more vigilant or anxious regarding FA, which may exaggerate the observed associations. Although the application of longitudinal trajectory modeling helps alleviate these issues by distinguishing persistent phenotypes from transient skin conditions, our results should be primarily interpreted as associations with “medically attended AD/FA” rather than strictly diagnosed conditions. Third, despite adjustment for various baseline covariates, the possibility of residual confounding from unmeasured factors cannot be ruled out. Specifically, information on parental history of allergic diseases was unavailable in this dataset. Fourth, the possibility of detection bias, particularly in the “Late-onset” group, cannot be ruled out. Parents of children with FA may be more vigilant regarding skin symptoms, and frequent visits for FA management increase the likelihood of AD/eczema diagnosis, possibly exaggerating the association between early FA and AD/eczema healthcare visit history. Fifth, the survey did not include allergic rhinitis as a selectable response option, preventing us from adjusting for this important atopic comorbidity or examining its role in the atopic march trajectory. Finally, this study was exclusively conducted in Japanese children. Owing to reports of higher FA diagnosis rates in Asian populations [23], the generalizability of our findings to other racial and ethnic groups warrants validation in diverse international cohorts.

In conclusion, this nationwide birth cohort study shows that distinct developmental AD/eczema trajectories are associated with FA risk. Although early-onset phenotypes confer the highest risk, consistent with the “atopic march,” elevated FA risk was observed across all groups, including “Late-onset” phenotypes, in which the onset of food allergy often preceded overt skin symptoms. These findings emphasize the importance of monitoring FA development in all children with AD/eczema, irrespective of disease onset or persistence.

## Supplementary Information

Below is the link to the electronic supplementary material.

Supplementary file1 (DOCX 13064 KB)

## Data Availability

The data that support the findings of this study are available from the Ministry of Health, Labour, and Welfare of Japan for the Longitudinal Survey of Newborns in the 21 st Century, but restrictions apply to the availability of these data, which were used under license for the current study, and so are not publicly available. However, data are available from the author upon reasonable request and with permission from the Ministry of Health, Labour, and Welfare of Japan.

## Abbreviations

AD: Atopic dermatitis
FA: Food allergy
GBTM: Group-based trajectory modeling
