# Supplementary material for: Atopic dermatitis/eczema phenotypes and their association with food allergy: a nationwide birth cohort study in Japan
Source: Eur J Pediatr. 2026 Jun 22;185(7):513. doi: 10.1007/s00431-026-07192-y (PMC13287158; doi:10.1007/s00431-026-07192-y)
Supplement: Supplementary file 1 — Supplementary file1 (DOCX 13064 KB) [file 431_2026_7192_MOESM1_ESM.docx]

**Supporting Information**

**Text S1. Selectable Response Options for Parent-Reported Healthcare Visits**

Parents were asked to select all applicable items from the following list when reporting their child's healthcare visits or medical history over the past year:

1. None

2. Pertussis

3. Varicella

4. Measles

5. Rubella

6. Exanthema subitum

7. Intussusception

8. Kawasaki disease

9. Allergic rhinitis or allergic conjunctivitis

10. Asthma

11. Atopic dermatitis

12. Food allergy

13. Conjunctivitis (excluding allergic)

14. Otitis media or otitis externa

15. Common cold, pharyngitis, tonsillitis, bronchitis, or pneumonia

16. Influenza

17. Gastrointestinal diseases such as gastroenteritis, diarrhea, abdominal pain, or constipation

18. Impetigo contagiosa

19. Eczema

20. Other types of dermatitis

21. Congenital diseases

22. Seizures or convulsions

23. Consultations regarding development and behavior

24. Dental caries

25. Other illnesses

26. Contusions or lacerations

27. Fractures

28. Burns

29. Other injuries

**Figure S1. Five identified trajectory phenotypes of atopic dermatitis/eczema**


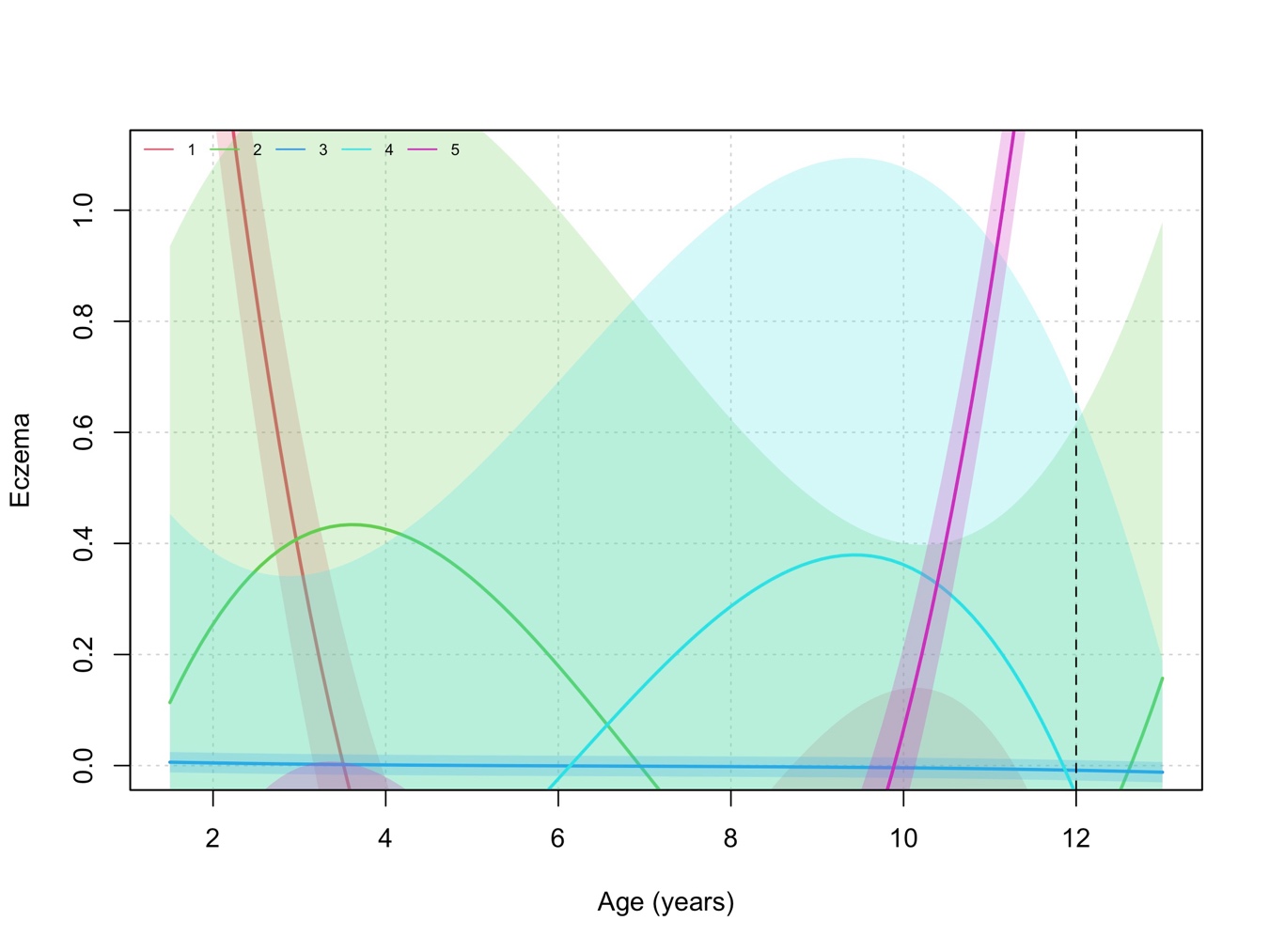


This figure illustrates the five distinct trajectory phenotypes of atopic dermatitis/eczema identified from age 1.5 to 12 years using group-based trajectory modeling. The solid lines depict the mean estimated probability for each phenotype group, while the shaded areas represent the 95% confidence intervals.

Trajectories are numbered and colored as follows:

(1) Red: Early-onset transient - High initial probability of healthcare visits that declines rapidly during early childhood.

(2) Green: Early-onset persistent - High initial probability that remains elevated throughout early childhood before gradually declining.

(3) Blue: No/minimal symptoms - A consistently near-zero probability of healthcare visits throughout the entire period.

(4) Cyan: Toddler-onset persistent - Low initial probability that rises to a peak around ages 3–4 and then slowly decreases.

(5) Purple: Late-onset - Very low probability during early and middle childhood, followed by a sharp increase in late childhood and early adolescence.

**Figure S2. Sensitivity analysis: Association of atopic dermatitis/eczema phenotypes with history of healthcare visit for food allergy (complete-case analysis)**

(A) Cumulative history of healthcare visit for food allergy from ages 0.5 to 12 years.


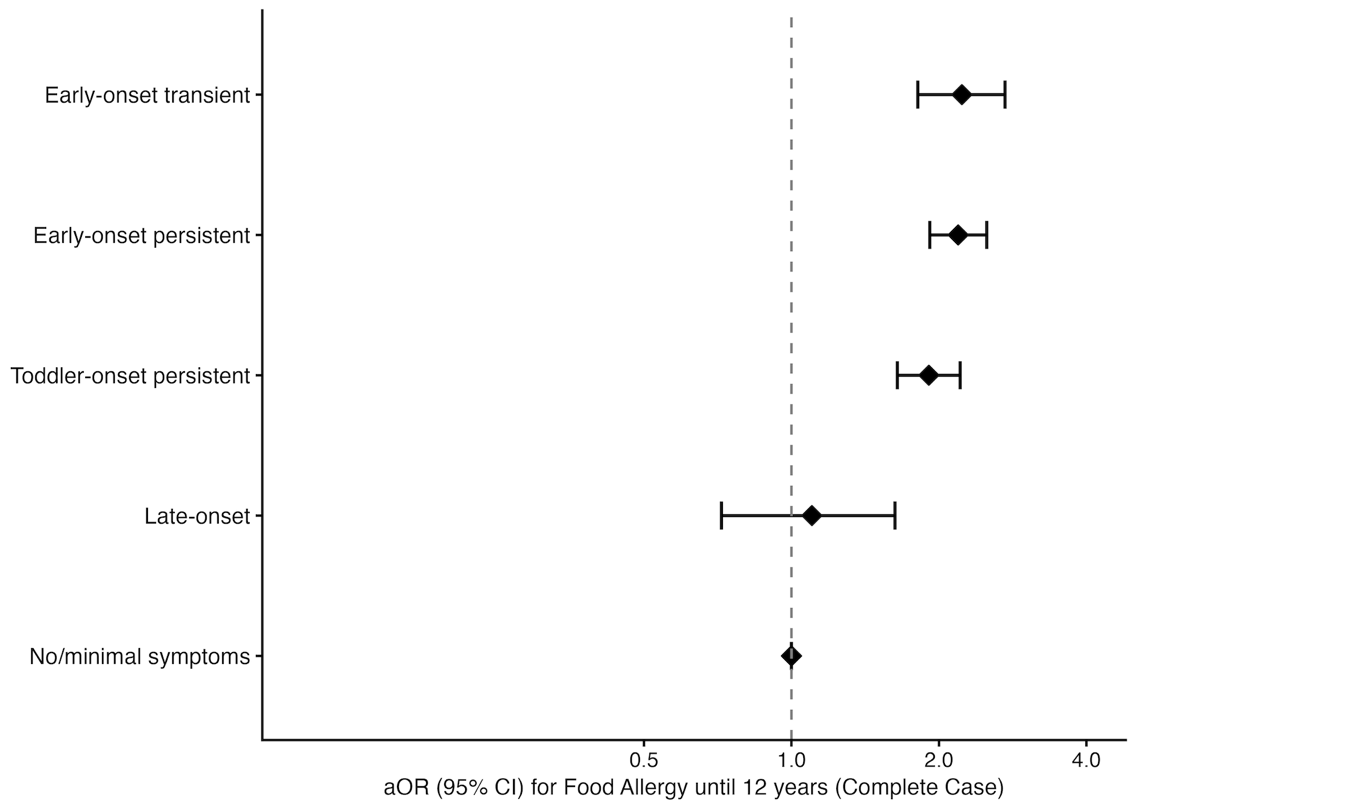


(B) Cumulative history of healthcare visit for food allergy during early childhood (ages 0.5–5.5 years).


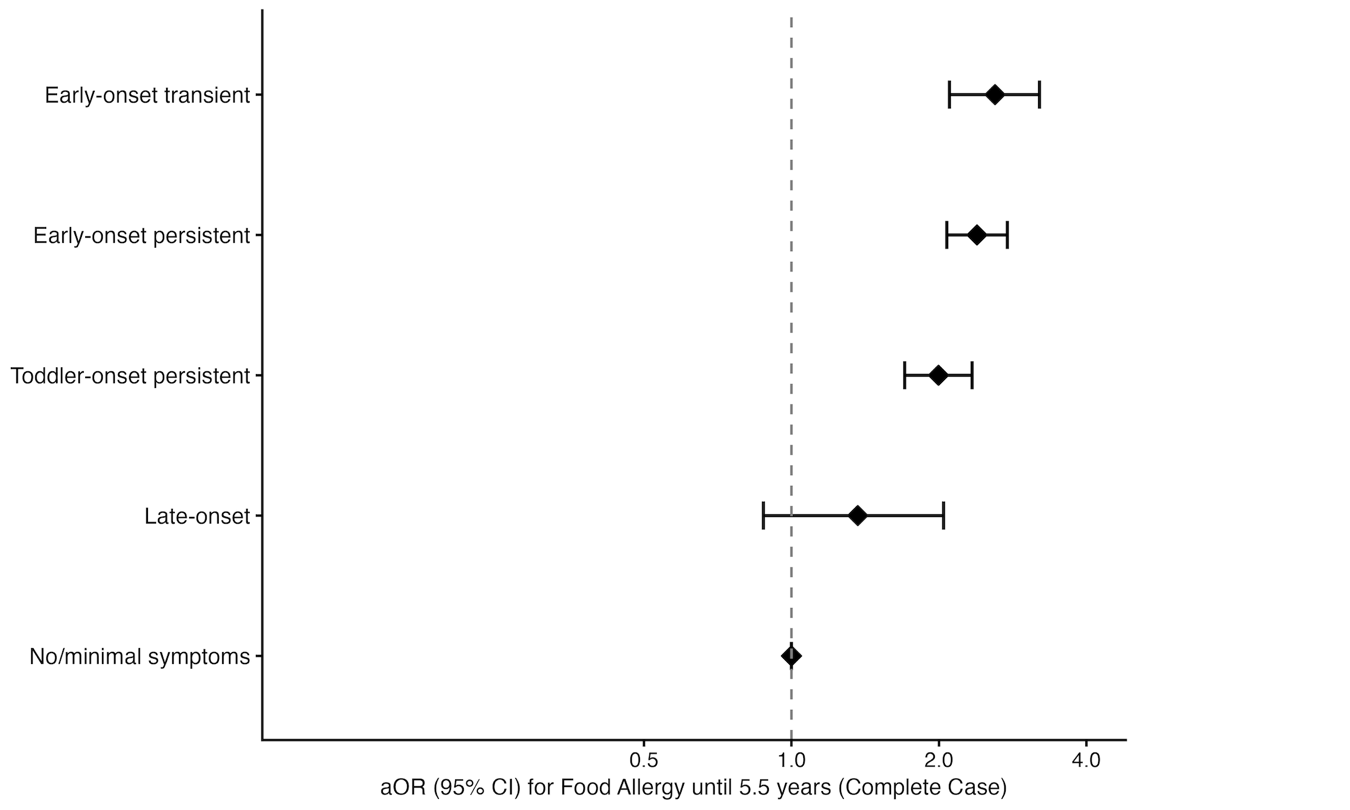


(C) Cumulative history of healthcare visit for food allergy during school age (ages 6–12 years).


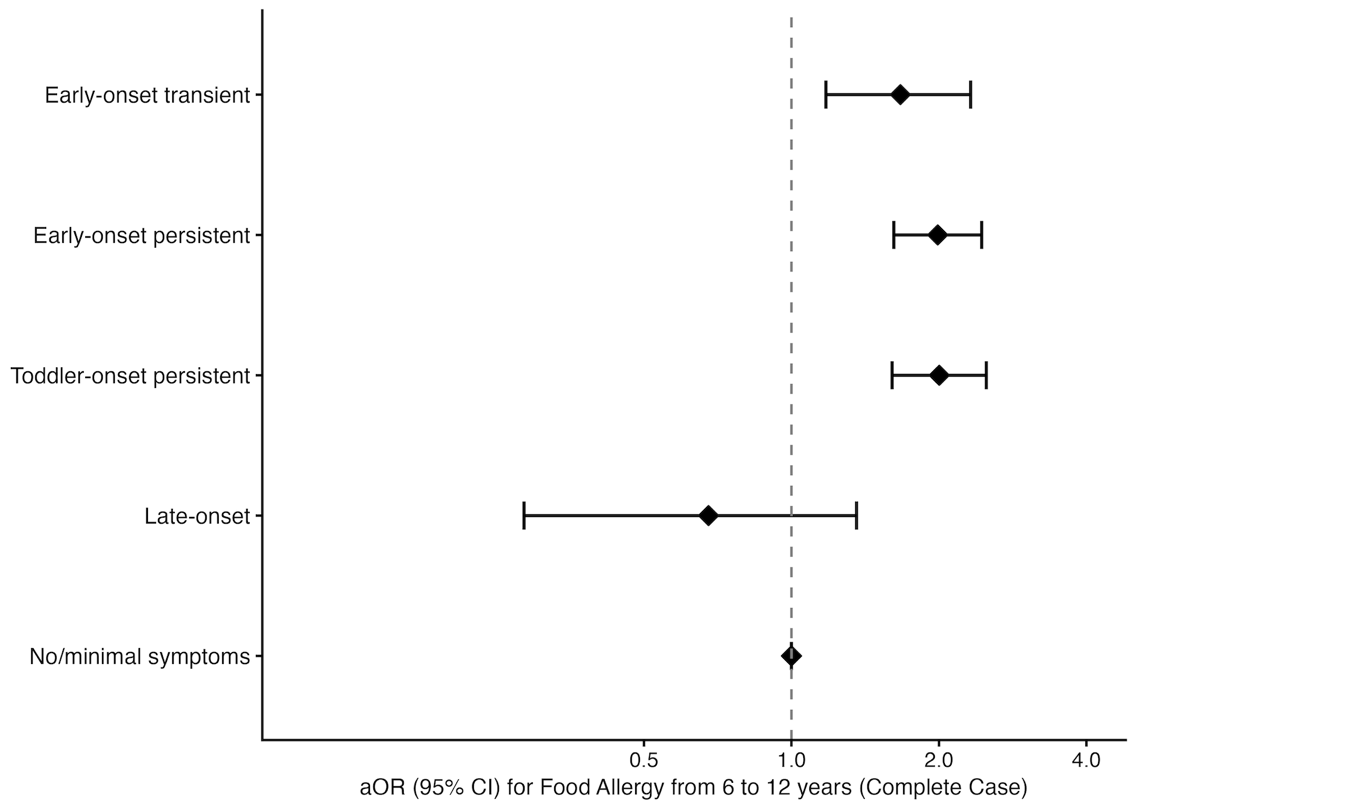


Forest plots display adjusted odds ratios (aORs) (points) and 95% confidence intervals (Cis) (horizontal lines) derived from multivariable logistic regression models. Data were handled using complete-case analysis, excluding participants with missing data. The “No/minimal symptoms” phenotype serves as the reference group. Models were adjusted for sociodemographic factors, perinatal characteristics, and cumulative history of healthcare visit for asthma. The plot represents the cumulative history of healthcare visit for food allergy during the entire follow-up period from ages 0.5 to 12 years.

**Table S1. Comparison of characteristics between analysed and excluded cases**

|  | **Overall**  **N = 38,554** | **Analysed cases**  **N = 23,767** | **Excluded cases**  **N = 14,787** | **p-value** |
| --- | --- | --- | --- | --- |
| **Sex** |  |  |  | 0.3 |
| Female | 18,710 (49%) | 11,487 (48%) | 7,223 (49%) |  |
| Male | 19,844 (51%) | 12,280 (52%) | 7,564 (51%) |  |
| **Birth weight** |  |  |  | 0.003 |
| <2500 g | 3,642 (9.4%) | 2,161 (9.1%) | 1,481 (10%) |  |
| ≥2500 g | 34,905 (91%) | 21,603 (91%) | 13,302 (90%) |  |
| Missing | 7 | 3 | 4 |  |
| **Gestational age** |  |  |  | <0.001 |
| <37 weeks | 2,098 (5.4%) | 1,215 (5.1%) | 883 (6.0%) |  |
| ≥37 weeks | 36,449 (95%) | 22,549 (95%) | 13,900 (94%) |  |
| Missing | 7 | 3 | 4 |  |
| **Number of elder siblings at 6 months** |  |  |  | 0.002 |
| ≥1 | 20,030 (52%) | 12,199 (51%) | 7,831 (53%) |  |
| 0 | 18,518 (48%) | 11,567 (49%) | 6,951 (47%) |  |
| Missing | 6 | 1 | 5 |  |
| **Birth type** |  |  |  | 0.001 |
| Multiple | 723 (1.9%) | 404 (1.7%) | 319 (2.2%) |  |
| Singleton | 37,831 (98%) | 23,363 (98%) | 14,468 (98%) |  |
| **Maternal smoking status at 6 months** |  |  |  | <0.001 |
| Nonsmoking | 35,757 (93%) | 22,760 (96%) | 12,997 (88%) |  |
| Smoking | 2,687 (7.0%) | 956 (4.0%) | 1,731 (12%) |  |
| Missing | 110 | 51 | 59 |  |
| **Paternal smoking status at 6 months** |  |  |  | <0.001 |
| Nonsmoking | 21,958 (58%) | 14,795 (63%) | 7,163 (50%) |  |
| Smoking | 15,678 (42%) | 8,590 (37%) | 7,088 (50%) |  |
| Missing | 918 | 382 | 536 |  |
| **Mother's Education** |  |  |  | <0.001 |
| Junior high school | 1,590 (4.8%) | 712 (3.1%) | 878 (8.7%) |  |
| High school | 15,454 (46%) | 10,106 (44%) | 5,348 (53%) |  |
| University/junior college/vocational school | 16,139 (48%) | 12,339 (53%) | 3,800 (38%) |  |
| Others | 94 (0.3%) | 64 (0.3%) | 30 (0.3%) |  |
| Missing | 5,277 | 546 | 4,731 |  |
| **Father's Education** |  |  |  | <0.001 |
| Junior high school | 2,157 (6.6%) | 1,151 (5.0%) | 1,006 (10%) |  |
| High school | 14,921 (46%) | 9,889 (43%) | 5,032 (51%) |  |
| University/junior college/vocational school | 15,519 (47%) | 11,815 (52%) | 3,704 (38%) |  |
| Others | 105 (0.3%) | 67 (0.3%) | 38 (0.4%) |  |
| Missing | 5,852 | 845 | 5,007 |  |
| **Daycare attendance at 6 months** |  |  |  | <0.001 |
| Attending | 1,361 (3.5%) | 734 (3.1%) | 627 (4.2%) |  |
| Not attending | 37,188 (96%) | 23,031 (97%) | 14,157 (96%) |  |
| Missing | 5 | 2 | 3 |  |
| **Household Income** |  |  |  | <0.001 |
| <2.50 million JPY | 1,758 (5.0%) | 787 (3.5%) | 971 (7.6%) |  |
| 2.50–4.99 million JPY | 12,984 (37%) | 7,500 (34%) | 5,484 (43%) |  |
| 5.00–7.49 million JPY | 12,294 (35%) | 8,213 (37%) | 4,081 (32%) |  |
| 7.50–9.99 million JPY | 5,005 (14%) | 3,596 (16%) | 1,409 (11%) |  |
| ≥10.00 million JPY | 2,931 (8.4%) | 2,130 (9.6%) | 801 (6.3%) |  |
| Missing | 3,582 | 1,541 | 2,041 |  |
| **Feeding practice** |  |  |  | <0.001 |
| Exclusive breastfeeding | 12,886 (34%) | 8,343 (35%) | 4,543 (31%) |  |
| Exclusive formula feeding | 1,345 (3.5%) | 698 (3.0%) | 647 (4.4%) |  |
| Mixed feeding | 23,988 (63%) | 14,599 (62%) | 9,389 (64%) |  |
| Missing | 335 | 127 | 208 |  |

**Table S2. Missing data patterns for atopic dermatitis/eczema healthcare visits across 11 follow-up surveys**

| **Age at Survey (years)** | **Missing Values, n (%)** |
| --- | --- |
| 1.5 | 6,519 (16.9) |
| 2.5 | 7,608 (19.7) |
| 3.5 | 10,381 (26.9) |
| 4.5 | 11,963 (31.0) |
| 5.5 | 12,025 (31.2) |
| 7 | 14,088 (36.5) |
| 8 | 14,805 (38.4) |
| 9 | 15,098 (39.2) |
| 10 | 15,395 (39.9) |
| 11 | 16,764 (43.5) |
| 12 | 18,028 (46.8) |

**Table S3 Comparison of characteristics between participants with and without food allergy data**^a^

|  | Available  N = 13,546 | Missing  N = 10,221 | *P*-value^b^ |
| --- | --- | --- | --- |
| **Sex** |  |  | 0.011 |
| Female | 6,450 (48%) | 5,037 (49%) |  |
| Male | 7,096 (52%) | 5,184 (51%) |  |
| **Birth weight** |  |  | 0.043 |
| <2,500 g | 1,276 (9.4%) | 885 (8.7%) |  |
| ≥2,500 g | 12,268 (91%) | 9,335 (91%) |  |
| Missing | 2 | 1 |  |
| **Gestational age** |  |  | 0.3 |
| <37 weeks | 711 (5.2%) | 504 (4.9%) |  |
| ≥37 weeks | 12,833 (95%) | 9,716 (95%) |  |
| Missing | 2 | 1 |  |
| **Number of elder siblings at 6 months** |  |  | 0.002 |
| ≥1 | 6,834 (50%) | 5,365 (52%) |  |
| 0 | 6,711 (50%) | 4,856 (48%) |  |
| Missing | 1 | 0 |  |
| **Birth type** |  |  | 0.5 |
| Multiple | 237 (1.7%) | 167 (1.6%) |  |
| Singleton | 13,309 (98%) | 10,054 (98%) |  |
| **Maternal smoking status at 6 months** |  |  | <0.001 |
| Nonsmoking | 13,088 (97%) | 9,672 (95%) |  |
| Smoking | 428 (3.2%) | 528 (5.2%) |  |
| Missing | 30 | 21 |  |
| **Paternal smoking status at 6 months** |  |  | <0.001 |
| Nonsmoking | 8,701 (65%) | 6,094 (61%) |  |
| Smoking | 4,650 (35%) | 3,940 (39%) |  |
| Missing | 195 | 187 |  |
| **Paternal educational attainment** |  |  | <0.001 |
| Junior high school | 569 (4.3%) | 582 (6.1%) |  |
| High school | 5,508 (41%) | 4,381 (46%) |  |
| University/junior college/vocational school | 7,211 (54%) | 4,604 (48%) |  |
| Others | 42 (0.3%) | 25 (0.3%) |  |
| Missing | 216 | 629 |  |
| **Maternal educational attainment** |  |  | <0.001 |
| Junior high school | 337 (2.5%) | 375 (3.9%) |  |
| High school | 5,546 (41%) | 4,560 (47%) |  |
| University/junior college/vocational school | 7,566 (56%) | 4,773 (49%) |  |
| Others | 40 (0.3%) | 24 (0.2%) |  |
| Missing | 57 | 489 |  |
| **Daycare attendance at 6 months** |  |  | 0.007 |
| Attending | 383 (2.8%) | 351 (3.4%) |  |
| Not attending | 13,162 (97%) | 9,869 (97%) |  |
| Missing | 1 | 1 |  |
| **Household Income** |  |  | <0.001 |
| <2.50 million JPY | 375 (2.9%) | 412 (4.4%) |  |
| 2.50–4.99 million JPY | 4,137 (32%) | 3,363 (36%) |  |
| 5.00–7.49 million JPY | 4,854 (38%) | 3,359 (36%) |  |
| 7.50–9.99 million JPY | 2,175 (17%) | 1,421 (15%) |  |
| ≥10.00 million JPY | 1,241 (9.7%) | 889 (9.4%) |  |
| Missing | 764 | 777 |  |
| **Feeding practice** |  |  | 0.002 |
| Exclusive breastfeeding | 4,713 (35%) | 3,630 (36%) |  |
| Exclusive formula feeding | 357 (2.6%) | 341 (3.4%) |  |
| Mixed feeding | 8,411 (62%) | 6,188 (61%) |  |
| Missing | 65 | 62 |  |
| **Asthma healthcare visit history** |  |  | <0.001 |
| No | 10,283 (80%) | 0 (0%) |  |
| Yes | 2,552 (20%) | 1,551 (100%) |  |
| Missing | 711 | 8,670 |  |
| **AD Trajectory Group** |  |  | <0.001 |
| Early-onset transient | 952 (7.0%) | 570 (5.6%) |  |
| Early-onset persistent | 3,505 (26%) | 1,999 (20%) |  |
| Toddler-onset persistent | 2,691 (20%) | 1,422 (14%) |  |
| Late-onset | 306 (2.3%) | 196 (1.9%) |  |
| No/minimal symptoms | 6,092 (45%) | 6,034 (59%) |  |

^a^Data are expressed as numbers (%) for categorical variables. ^b^*P* values were calculated to evaluate differences across the AD/eczema phenotypes using the chi-squared test for categorical variables.

**Table S4. Baseline characteristics of the imputed dataset according to atopic dermatitis/eczema phenotypes^a^**

| Characteristic | Overall, N = 23,767 | Early-onset transient, N = 1,522 | Early-onset persistent, N = 5,504 | Toddler-onset persistent, N = 4,113 | Late-onset, N = 502 | No/minimal symptoms, N = 12,126 | *P*-value^b^ |
| --- | --- | --- | --- | --- | --- | --- | --- |
| **Sex** |  |  |  |  |  |  | <0.001 |
| Female | 11,487 (48%) | 683 (45%) | 2,660 (48%) | 2,087 (51%) | 254 (51%) | 5,803 (48%) |  |
| Male | 12,280 (52%) | 839 (55%) | 2,844 (52%) | 2,026 (49%) | 248 (49%) | 6,323 (52%) |  |
| **Birth weight** |  |  |  |  |  |  | <0.001 |
| <2,500 g | 2,162 (9.1%) | 116 (7.6%) | 520 (9.4%) | 373 (9.1%) | 48 (9.6%) | 1,105 (9.1%) |  |
| ≥2,500 g | 21,605 (91%) | 1,406 (92%) | 4,984 (91%) | 3,740 (91%) | 454 (90%) | 11,021 (91%) |  |
| **Gestational age** |  |  |  |  |  |  | <0.001 |
| <37 weeks | 1,215 (5.1%) | 67 (4.4%) | 296 (5.4%) | 200 (4.9%) | 24 (4.8%) | 628 (5.2%) |  |
| ≥37 weeks | 22,552 (95%) | 1,455 (96%) | 5,208 (95%) | 3,913 (95%) | 478 (95%) | 11,498 (95%) |  |
| **Number of elder siblings at 6 months** |  |  |  |  |  |  | <0.001 |
| ≥1 | 12,199 (51%) | 673 (44%) | 2,753 (50%) | 2,075 (50%) | 268 (53%) | 6,430 (53%) |  |
| 0 | 11,568 (49%) | 849 (56%) | 2,751 (50%) | 2,038 (50%) | 234 (47%) | 5,696 (47%) |  |
| **Birth type** |  |  |  |  |  |  | <0.001 |
| Multiple | 404 (1.7%) | 25 (1.6%) | 91 (1.7%) | 61 (1.5%) | 7 (1.4%) | 220 (1.8%) |  |
| Singleton | 23,363 (98%) | 1,497 (98%) | 5,413 (98%) | 4,052 (99%) | 495 (99%) | 11,906 (98%) |  |
| **Maternal smoking status at 6 months** |  |  |  |  |  |  | <0.001 |
| Nonsmoking | 22,807 (96%) | 1,468 (96%) | 5,314 (97%) | 3,952 (96%) | 485 (97%) | 11,587 (96%) |  |
| Smoking | 960 (4.0%) | 54 (3.5%) | 190 (3.4%) | 161 (3.9%) | 17 (3.4%) | 539 (4.4%) |  |
| **Paternal smoking status at 6 months** |  |  |  |  |  |  | <0.001 |
| Nonsmoking | 15,002 (63%) | 975 (64%) | 3,545 (64%) | 2,614 (64%) | 314 (62%) | 7,554 (62%) |  |
| Smoking | 8,765 (37%) | 547 (36%) | 1,959 (36%) | 1,499 (36%) | 188 (38%) | 4,572 (38%) |  |
| **Paternal educational attainment** |  |  |  |  |  |  | <0.001 |
| Junior high school | 1,222 (5.1%) | 56 (3.7%) | 245 (4.4%) | 231 (5.6%) | 22 (4.5%) | 668 (5.5%) |  |
| High school | 10,303 (43%) | 623 (41%) | 2,277 (41%) | 1,722 (42%) | 216 (43%) | 5,465 (45%) |  |
| University/junior college/vocational school | 12,172 (51%) | 838 (55%) | 2,965 (54%) | 2,152 (52%) | 261 (52%) | 5,957 (49%) |  |
| Others | 70 (0.3%) | 4 (0.3%) | 18 (0.3%) | 8 (0.2%) | 3 (0.6%) | 36 (0.3%) |  |
| **Maternal educational attainment** |  |  |  |  |  |  | <0.001 |
| Junior high school | 737 (3.1%) | 44 (2.9%) | 159 (2.9%) | 126 (3.1%) | 10 (1.9%) | 399 (3.3%) |  |
| High school | 10,363 (44%) | 602 (40%) | 2,244 (41%) | 1,716 (42%) | 222 (44%) | 5,580 (46%) |  |
| University/junior college/vocational school | 12,601 (53%) | 876 (58%) | 3,082 (56%) | 2,257 (55%) | 271 (54%) | 6,116 (50%) |  |
| Others | 66 (0.3%) | 0 (0%) | 20 (0.4%) | 14 (0.3%) | 0 (0%) | 32 (0.3%) |  |
| **Daycare attendance at 6 months** |  |  |  |  |  |  | <0.001 |
| Attending | 734 (3.1%) | 53 (3.5%) | 173 (3.1%) | 112 (2.7%) | 12 (2.4%) | 384 (3.2%) |  |
| Not attending | 23,033 (97%) | 1,469 (97%) | 5,331 (97%) | 4,001 (97%) | 490 (98%) | 11,742 (97%) |  |
| **Household Income** |  |  |  |  |  |  | <0.001 |
| <2.50 million JPY | 859 (3.6%) | 48 (3.2%) | 146 (2.7%) | 135 (3.3%) | 18 (3.6%) | 512 (4.2%) |  |
| 2.50–4.99 million JPY | 8,084 (34%) | 501 (33%) | 1,764 (32%) | 1,419 (34%) | 159 (32%) | 4,241 (35%) |  |
| 5.00–7.49 million JPY | 8,764 (37%) | 562 (37%) | 2,050 (37%) | 1,501 (36%) | 195 (39%) | 4,456 (37%) |  |
| 7.50–9.99 million JPY | 3,812 (16%) | 263 (17%) | 946 (17%) | 686 (17%) | 78 (16%) | 1,839 (15%) |  |
| ≥10.00 million JPY | 2,248 (9.5%) | 149 (9.8%) | 598 (11%) | 373 (9.1%) | 51 (10%) | 1,077 (8.9%) |  |
| **Feeding practice** |  |  |  |  |  |  | <0.001 |
| Exclusive breastfeeding | 8,390 (35%) | 520 (34%) | 1,953 (35%) | 1,414 (34%) | 198 (39%) | 4,306 (36%) |  |
| Exclusive formula feeding | 702 (3.0%) | 54 (3.6%) | 127 (2.3%) | 101 (2.4%) | 9 (1.8%) | 411 (3.4%) |  |
| Mixed feeding | 14,675 (62%) | 948 (62%) | 3,424 (62%) | 2,599 (63%) | 295 (59%) | 7,409 (61%) |  |
| **Food allergy healthcare visit history** | 2,640 (11%) | 236 (16%) | 868 (16%) | 624 (15%) | 51 (10%) | 861 (7.1%) | <0.001 |
| **Asthma healthcare visit history** | 4,289 (18%) | 267 (18%) | 1,144 (21%) | 882 (21%) | 102 (20%) | 1,893 (16%) | <0.001 |

^a^Data are expressed as numbers (%) for categorical variables. ^b^*P* values were calculated to evaluate differences across the AD/eczema phenotypes using the chi-squared test for categorical variables.

**Table S5.** Goodness-of-fit statistics for group-based trajectory models of atopic dermatitis/eczema healthcare visits

|  | BIC | AIC | Smallest APP |
| --- | --- | --- | --- |
| **Linear** |  |  |  |
| 1-cluster | 511597.1 | 511565.7 | 1.00 |
| 2-cluster | -390623.4 | -390696.7 | 1.00 |
| 3-cluster | -392536.5 | -392651.7 | 0.8460067 |
| 4-cluster | -391535.6 | -391692.7 | 0.5235115 |
| 5-cluster | -392504.2 | -392703.2 | NaN |
| **Quadratic** |  |  |  |
| 1-cluster | 511411.5 | 511369.6 | 1.00 |
| 2-cluster | -410051 | -410146.1 | 1.00 |
| 3-cluster | -415297.8 | -415444.5 | 0.8946902 |
| 4-cluster | -417444.6 | -417643.6 | 0.8712523 |
| 5-cluster | -417846.3 | -418097.7 | 0.6753368 |
| **Cubic** |  |  |  |
| 1-cluster | 511413.5 | 511361.1 | 1.00 |
| 2-cluster | -407020.6 | -407135.8 | 1.00 |
| 3-cluster | -419053.1 | -419231.1 | 0.9807368 |
| 4-cluster | -420667.4 | -420908.3 | 0.8596933 |
| 5-cluster | -421193.3 | -421497.1 | 0.8594082 |

Note: The table presents goodness-of-fit statistics for models with a varying number of trajectory groups (1 to 5 clusters) and different polynomial functional forms (linear, quadratic, and cubic) . The optimal model was determined based on the lowest Bayesian Information Criterion (BIC) value, an average posterior probability (APP) of ≥0.80 for each group, sufficient group size, and clinical interpretability. Based on these criteria, the five-group cubic polynomial model was selected as the best-fitting model.

Abbreviations: AIC, Akaike Information Criterion; APP, Average Posterior Probability; BIC, Bayesian Information Criterion.

**Table S6. Baseline characteristics in complete-case analysis according to atopic dermatitis/eczema phenotypes^a^**

|  | Overall  N = 11,901 | Early-onset transient  N = 827 | Early-onset persistent  N = 3,095 | Toddler-onset persistent  N = 2,326 | Late-onset  N = 268 | No/minimal symptoms N = 5,385 | *P*-value^b^ |
| --- | --- | --- | --- | --- | --- | --- | --- |
| **Sex** |  |  |  |  |  |  | <0.001 |
| Female | 5,679 (48%) | 347 (42%) | 1,476 (48%) | 1,189 (51%) | 137 (51%) | 2,530 (47%) |  |
| Male | 6,222 (52%) | 480 (58%) | 1,619 (52%) | 1,137 (49%) | 131 (49%) | 2,855 (53%) |  |
| **Birth weight** |  |  |  |  |  |  | 0.7 |
| <2,500 g | 1,129 (9.5%) | 78 (9.4%) | 306 (9.9%) | 216 (9.3%) | 31 (12%) | 498 (9.2%) |  |
| ≥2,500 g | 10,772 (91%) | 749 (91%) | 2,789 (90%) | 2,110 (91%) | 237 (88%) | 4,887 (91%) |  |
| **Gestational age** |  |  |  |  |  |  | 0.6 |
| <37 weeks | 623 (5.2%) | 42 (5.1%) | 171 (5.5%) | 108 (4.6%) | 12 (4.5%) | 290 (5.4%) |  |
| ≥37 weeks | 11,278 (95%) | 785 (95%) | 2,924 (94%) | 2,218 (95%) | 256 (96%) | 5,095 (95%) |  |
| **Number of elder siblings at 6 months** |  |  |  |  |  |  | <0.001 |
| ≥1 | 6,027 (51%) | 349 (42%) | 1,530 (49%) | 1,150 (49%) | 140 (52%) | 2,858 (53%) |  |
| 0 | 5,874 (49%) | 478 (58%) | 1,565 (51%) | 1,176 (51%) | 128 (48%) | 2,527 (47%) |  |
| **Birth type** |  |  |  |  |  |  | 0.4 |
| Multiple | 210 (1.8%) | 11 (1.3%) | 54 (1.7%) | 33 (1.4%) | 4 (1.5%) | 108 (2.0%) |  |
| Singleton | 11,691 (98%) | 816 (99%) | 3,041 (98%) | 2,293 (99%) | 264 (99%) | 5,277 (98%) |  |
| **Maternal smoking status at 6 months** |  |  |  |  |  |  | 0.4 |
| Nonsmoking | 11,564 (97%) | 805 (97%) | 3,021 (98%) | 2,261 (97%) | 261 (97%) | 5,216 (97%) |  |
| Smoking | 337 (2.8%) | 22 (2.7%) | 74 (2.4%) | 65 (2.8%) | 7 (2.6%) | 169 (3.1%) |  |
| **Paternal smoking status at 6 months** |  |  |  |  |  |  | 0.11 |
| Nonsmoking | 7,799 (66%) | 544 (66%) | 2,052 (66%) | 1,540 (66%) | 157 (59%) | 3,506 (65%) |  |
| Smoking | 4,102 (34%) | 283 (34%) | 1,043 (34%) | 786 (34%) | 111 (41%) | 1,879 (35%) |  |
| **Paternal educational attainment** |  |  |  |  |  |  |  |
| Junior high school | 478 (4.0%) | 28 (3.4%) | 113 (3.7%) | 104 (4.5%) | 11 (4.1%) | 222 (4.1%) |  |
| High school | 4,851 (41%) | 307 (37%) | 1,239 (40%) | 939 (40%) | 112 (42%) | 2,254 (42%) |  |
| University/junior college/vocational school | 6,534 (55%) | 491 (59%) | 1,734 (56%) | 1,277 (55%) | 145 (54%) | 2,887 (54%) |  |
| Others | 38 (0.3%) | 1 (0.1%) | 9 (0.3%) | 6 (0.3%) | 0 (0%) | 22 (0.4%) |  |
| **Maternal educational attainment** |  |  |  |  |  |  |  |
| Junior high school | 262 (2.2%) | 16 (1.9%) | 62 (2.0%) | 56 (2.4%) | 5 (1.9%) | 123 (2.3%) |  |
| High school | 4,776 (40%) | 294 (36%) | 1,199 (39%) | 883 (38%) | 116 (43%) | 2,284 (42%) |  |
| University/junior college/vocational school | 6,829 (57%) | 517 (63%) | 1,821 (59%) | 1,377 (59%) | 147 (55%) | 2,967 (55%) |  |
| Others | 34 (0.3%) | 0 (0%) | 13 (0.4%) | 10 (0.4%) | 0 (0%) | 11 (0.2%) |  |
| **Daycare attendance at 6 months** |  |  |  |  |  |  | 0.3 |
| Attending | 320 (2.7%) | 27 (3.3%) | 78 (2.5%) | 50 (2.1%) | 8 (3.0%) | 157 (2.9%) |  |
| Not attending | 11,581 (97%) | 800 (97%) | 3,017 (97%) | 2,276 (98%) | 260 (97%) | 5,228 (97%) |  |
| **Household Income** |  |  |  |  |  |  | 0.3 |
| <2.50 million JPY | 339 (2.8%) | 23 (2.8%) | 71 (2.3%) | 71 (3.1%) | 7 (2.6%) | 167 (3.1%) |  |
| 2.50–4.99 million JPY | 3,843 (32%) | 249 (30%) | 958 (31%) | 763 (33%) | 86 (32%) | 1,787 (33%) |  |
| 5.00–7.49 million JPY | 4,523 (38%) | 322 (39%) | 1,188 (38%) | 855 (37%) | 108 (40%) | 2,050 (38%) |  |
| 7.50–9.99 million JPY | 2,042 (17%) | 151 (18%) | 564 (18%) | 408 (18%) | 38 (14%) | 881 (16%) |  |
| ≥10.00 million JPY | 1,154 (9.7%) | 82 (9.9%) | 314 (10%) | 229 (9.8%) | 29 (11%) | 500 (9.3%) |  |
| **Feeding practice** |  |  |  |  |  |  | 0.002 |
| Exclusive breastfeeding | 4,150 (35%) | 260 (31%) | 1,099 (36%) | 809 (35%) | 102 (38%) | 1,880 (35%) |  |
| Exclusive formula feeding | 307 (2.6%) | 25 (3.0%) | 58 (1.9%) | 47 (2.0%) | 4 (1.5%) | 173 (3.2%) |  |
| Mixed feeding | 7,444 (63%) | 542 (66%) | 1,938 (63%) | 1,470 (63%) | 162 (60%) | 3,332 (62%) |  |
| **Asthma healthcare visit history** | 2,309 (19%) | 161 (19%) | 690 (22%) | 523 (22%) | 63 (24%) | 872 (16%) | <0.001 |
| **Food allergy healthcare visit history** | 1,641 (14%) | 154 (19%) | 582 (19%) | 394 (17%) | 29 (11%) | 482 (9.0%) | <0.001 |

^a^Data are expressed as numbers (%) for categorical variables. ^b^*P* values were calculated to evaluate differences across the AD/eczema phenotypes using the chi-squared test for categorical variables.

AD, atopic dermatitis; FA, food allergy

*Note*: Complete-case analysis included 11,901 participants (50% of the imputed dataset). The Late-onset group experienced the largest proportional reduction (83%), which likely contributed to reduced statistical power. Lower prevalence of food allergy

(14% vs 18.5%) and asthma (19% vs 28.5%) in the complete-case dataset suggests potential selection bias toward healthier participants."
